# Comparative efficacy of various exercise therapies for chronic fatigue syndrome: A systematic review and network meta-analysis

**DOI:** 10.1016/j.isci.2025.114178

**Published:** 2025-11-21

**Authors:** Zhongxin Liao, Suhong Zhao, Sitong Fang, Jun Ren, Shoujian Wang, Lingjun Kong, Min Fang

**Affiliations:** 1 Department of Tuina, Shuguang Hospital, Shanghai University of Traditional Chinese Medicine, Shanghai 201203, China; 2 Institute of Tuina, Shanghai Institute of Traditional Chinese Medicine, Shanghai 201203, China

**Keywords:** human physiology, therapy, treatment

## Abstract

To systematically compare the effectiveness of different exercise therapies for chronic fatigue syndrome (CFS), we conducted a systematic review and network meta-analysis of randomized controlled trials based on searches of nine databases up to February 19, 2025. The review included 25 randomized controlled trials, with 20 trials (*n* = 2,831) eligible for network meta-analysis. Graded exercise therapy (GET) showed relatively superior short-term effects on fatigue (mean difference [MD]: −6.93, 95% confidence interval [CI]: −10.85 to −3.01; moderate certainty), depression (MD: −5.27, 95% CI: −7.38 to −3.16; low certainty), and anxiety (MD: −2.88, 95% CI: −5.10 to −0.66, low certainty) compared with waitlist at the end of treatments, with partial maintenance of effects at follow-up. Other modalities, including Qigong, Yoga, strength/resistance training, and running showed modest benefits but failed to surpass the minimally important difference with confidence, and were supported by low/very low certainty evidence. These findings support the short-term utility of GET in managing CFS symptoms. However, its broader clinical endorsement remains controversial, highlighting the need for further high-quality trials.

## Highlights

• Graded exercise therapy showed moderate-certainty short-term benefit in fatigue
• Other exercise modalities showed possible benefit with low/very low evidence certainty
• Long-term effects remain uncertain
• Findings highlight the need for individualized exercise and rigorous future trials

## Graphical abstract

**Figure:**
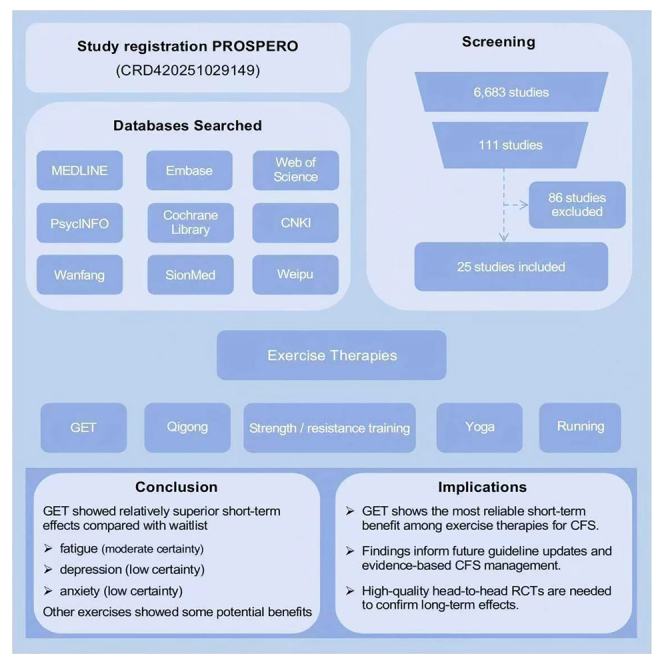

## Introduction

Chronic fatigue syndrome (CFS) is a disabling condition marked by persistent fatigue lasting at least 6 months, often accompanied by cognitive impairment, sleep disturbances, mood changes, and widespread pain.^1^^,^^2^^,^^3^ Its symptom profile overlaps with other chronic, multisystem conditions such as fibromyalgia and post-COVID condition, which share similar challenges in management and research.^4^^,^^5^ Affecting about 0.89% of the global population and associated with substantial economic burden, CFS severely impairs quality of life and productivity.^6^^,^^7^ To date, there are no approved pharmacological treatments for CFS, and current clinical strategies remain largely supportive and symptom-focused.^8^^,^^9^^,^^10^ Among non-pharmacological interventions, cognitive behavioral therapy (CBT) has shown the most evidence for limited efficacy in reducing fatigue, especially in non-severe patients.^11^ However, CBT-induced improvements are often not accompanied by increases in physical activity, suggesting behavioral adaptation rather than true functional recovery.^12^ Nutritional supplement has also been explored, but inconsistent results and methodological limitations preclude firm conclusions.^13^ Adaptive pacing therapy, a patient-centered approach, may help individuals manage symptoms, yet its effectiveness remains uncertain due to a lack of robust supporting evidence.^14^ By contrast, exercise therapy (ET) directly targets physical deconditioning and is one of the most widely recommended treatments for CFS. The Cochrane systematic review indicated that ET may offer broader benefits across fatigue, physical function, and self-perceived general health.^15^ Common modalities of ET for managing CFS include strength/resistance training, graded exercise therapy (GET), Qigong, Yoga, etc. Accumulating evidence suggests that exercise interventions may yield multidimensional benefits for patients with CFS. In addition to reducing fatigue severity, recent randomized controlled trials (RCTs) have demonstrated that ET significantly improves physical function, sleep quality, and emotional well-being.^16^^,^^17^^,^^18^^,^^19^ Furthermore, preliminary evidence suggests that ET may exert beneficial effects through modulation of inflammatory cytokines (with altered levels of IL-6, IL-8, and TNF-α), strengthening the functional connectivity of the left frontoparietal network and default mode network, and promoting the functional plasticity of brain networks in patients with CFS by regulating the information transmission between them.^20^^,^^21^ However, these mechanisms remain under investigation and require further elucidation in mechanistic studies. Although previous systematic reviews have primarily focused on comparing ET with other active treatments, such as CBT or pharmacologic interventions,^22^^,^^23^^,^^24^ comparative evidence on the relative effectiveness of different ET modalities for CFS remains insufficient. This limits clinical decision-making, as it remains unclear which specific modality, such as GET, resistance training, Qigong, or Yoga, provides the greatest benefit. To address this gap, we conducted a network meta-analysis (NMA), which synthesizes both direct and indirect evidence across RCTs. This method allows for evaluating and comparing the effectiveness of various ETs for CFS, even in the absence of head-to-head trials.^25^ By evaluating a wide range of ET, this study aims to provide robust, evidence-based guidance to inform individualized treatment strategies for CFS.

## Results

**Figure 1:**
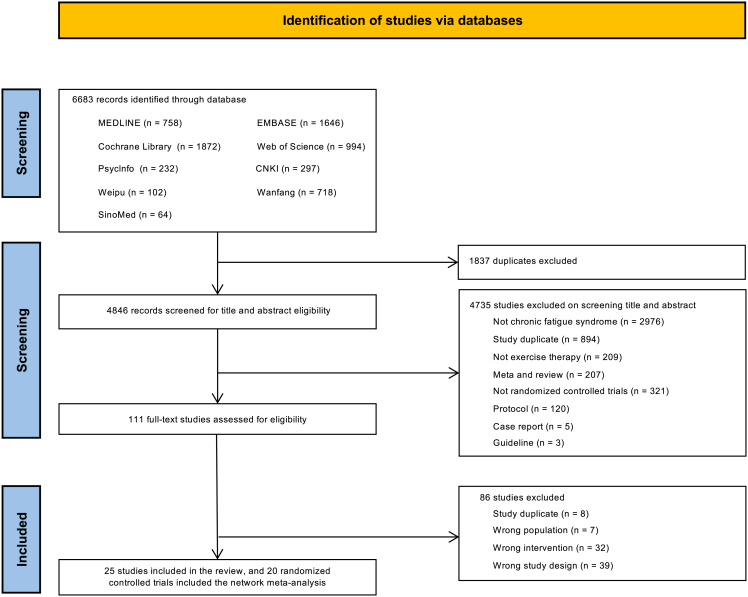
PRISMA flow diagram for systematic review

A total of 6,683 records were identified through database searches, of which 111 full-text articles were assessed for eligibility after title and abstract screening. Ultimately, 25 RCTs^16^^,^^18^^,^^21^^,^^26^^,^^27^^,^^28^^,^^29^^,^^30^^,^^31^^,^^32^^,^^33^^,^^34^^,^^35^^,^^36^^,^^37^^,^^38^^,^^39^^,^^40^^,^^41^^,^^42^^,^^43^^,^^44^^,^^45^^,^^46^^,^^47^ involving 3,602 patients with CFS were included. Of these, 20 RCTs^16^^,^^18^^,^^26^^,^^29^^,^^30^^,^^31^^,^^32^^,^^33^^,^^34^^,^^35^^,^^36^^,^^37^^,^^38^^,^^39^^,^^41^^,^^42^^,^^43^^,^^44^^,^^46^^,^^48^ comprising 2,831 participants were included in the network meta-analysis. The PRISMA flow diagram depicts the search and selection process (Figure 1). A comprehensive list of eligible studies is provided in Table 1.

**Table 1:** Characteristics of included studies

Study ID	Country	Diagnostic criteria	Sample size	Mean age (years)	Female (%)	Duration of illness (weeks)	Intervention vs. control	Duration of intervention (weeks)	Follow-up (weeks)	Outcomes
Chan et al.^36^	China	CDC	137	42.0	76.6	NR	QIGO vs. WAIT	17	NR	Fatigue, anxiety, depression
Chan et al.^30^	China	CDC	150	39.0	72.0	NR	QIGO vs. WAIT	9	NR	Fatigue, anxiety, physical function, depression, sleep
Chan et al.^29^	China	CDC	108	40.0	100	NR	QIGO vs. WAIT	9	12	Anxiety, depression
Chen et al.^26^	China	CDC	70	51.0	47.1	NR	QIGO vs. SMC	4	NR	Fatigue
Clark et al.^18^	UK	NICE	211	38.0	79.1	236	GET vs. WAIT	8	4	Fatigue, physical function
Clark et al.^21^	UK	NICE	211	38.0	79.1	238	GET vs. WAIT	8	40	Fatigue, physical function
Fulcher and White^32^	UK	Oxford	66	37.0	74.0	130	GET vs REFL	12	48	Fatigue, Anxiety, depression, sleep
Ho et al.^34^	China	CDC	64	42.0	79.7	NR	QIGO vs. WAIT	12	NR	Fatigue, physical function
Jason et al.^35^	USA	CDC	114	43.8	83.0	NR	STRE vs. REFL	24	48	Anxiety, depression
Moss-Morris et al.^37^	New Zealand	CDC	49	41.0	69.4	182	GET vs. SMC	12	30	Fatigue, physical function
Oka et al.^38^	Japan	CDC	30	39.0	80.0	NR	YOGA vs. WAIT	8	NR	Fatigue
Powell et al.^39^	UK	Oxford	148	33.0	78.4	NR	GET vs. SMC	12	36	Fatigue, physical function, sleep, anxiety, depression
Sutcliffe et al.^42^	UK	CDC	38	48.0	81.6	NR	STRE vs. REFL	24	NR	Fatigue
Wallman et al.^48^	Australia	CDC	61	NR	77.1	NR	GET vs REFL	12	NR	Fatigue, depression, anxiety
Wearden et al.^44^	UK	Oxford	68	NR	NR	NR	GET vs WAIT	26	NR	Fatigue
Wearden et al.^43^	UK	Oxford	296	45.0	77.7	NR	GET vs SMC	18	50	Fatigue, physical function, depression, anxiety, sleep
White et al.^16^	UK	Oxford	302	NR	NR	129	GET vs. SMC	24	48	Fatigue, physical function, depression, anxiety, sleep
Zhao et al.^46^	China	CDC	46	18.0	0	NR	RUN vs. WAIT	12	NR	Fatigue
Gordon et al.^33^	Australia	CDC	22	15.9	NR	NR	GET vs. STRE	4	NR	Fatigue, depression
Sharpe et al.^41^	UK	Oxford	640	39.0	77.30	129	GET vs. SMC	24	110	Fatigue, physical function
White et al.^45^	UK	Oxford	640	38	77.0	32	GET vs. SMC	52	NR	Fatigue, physical function
Wallman et al.^47^	Australia	CDC	68	NR	NR	NR	GET vs. REFL	12	NR	NR
Broadbent and Coutts^27^	Australia	CDC	24	50.9	41.2	151	GET vs. WAIT	12	NR	NR
Broadbent and Coutts^28^	Australia	CDC	24	50.9	41.2	151	GET vs. WAIT	12	NR	NR
Sandlar et al.^40^	Australia	CDC	15	32.1	40.0	234	Cycling vs. STRE	6	NR	Fatigue, pain, sleep, physical function

NR, not reported. All included trials applied recognized diagnostic criteria for CFS (e.g., CDC-1994, NICE-2021, or Oxford), which require symptoms to persist for a minimum of 3–6 months depending on the definition used. GET, graded exercise therapy; STRE, strength/resistance training; RUN, running; QIGO, qigong; YOGA, yoga; SMC, specialist medical care; REFL, relaxation/flexibility; WAIT, waitlist.

**Figure 2:**
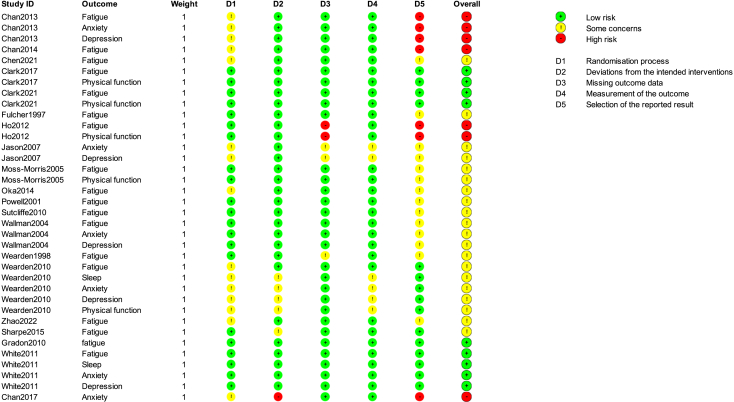
Risk of bias summary across outcomes Green, low risk of bias; Yellow, some concerns; Red, high risk of bias. Domains assessed include: (D1) randomization process, (D2) deviations from the intended interventions, (D3) missing outcome data, (D4) measurement of the outcome, and (D5) selection of the reported result.

Geographically, nearly half of the trials (40%, *n* = 10)^16^^,^^18^^,^^31^^,^^32^^,^^39^^,^^41^^,^^42^^,^^43^^,^^44^^,^^45^ were conducted in Europe, followed by 7 (28%)^26^^,^^29^^,^^30^^,^^34^^,^^36^^,^^38^^,^^46^ in Asia, 7 (28%)^27^^,^^28^^,^^37^^,^^47^^,^^48^ in Oceania, and 1 (4%)^35^ in North America. The median age of participants was 39.0 years (interquartile range [IQR]: 38.0–43.8), with a median female proportion of 77.2% (IQR: 71.4%–79.3%). The median duration of illness was 151.0 weeks (IQR: 129.3–221.0), and the median treatment duration was 12.0 weeks (IQR: 9.0–18.0). Ten trials included post-treatment follow-up, with a median duration of 44 weeks (IQR: 31.5–48.0). Among the 20 trials^18^^,^^29^^,^^30^^,^^31^^,^^32^^,^^33^^,^^34^^,^^35^^,^^36^^,^^37^^,^^38^^,^^39^^,^^41^^,^^42^ included in the NMA, 11 (55%)^16^^,^^18^^,^^31^^,^^32^^,^^33^^,^^37^^,^^39^^,^^41^^,^^44^^,^^48^ evaluated GET, 5 (25%)^26^^,^^29^^,^^30^^,^^34^^,^^36^ assessed Qigong, 2 (10%)^35^^,^^42^ evaluated strength/resistance training, and 1 trial each (5%) investigated Yoga^38^ and running.^46^ Control interventions included waitlist (*n* = 13),^16^^,^^18^^,^^29^^,^^30^^,^^31^^,^^34^^,^^36^^,^^37^^,^^38^^,^^39^^,^^41^^,^^44^^,^^46^ relaxation or flexibility exercises (*n* = 4),^32^^,^^35^^,^^42^^,^^48^ specialist medical care (*n* = 2),^26^^,^^43^ and strength/resistance training (*n* = 1).^33^ High risk of bias most frequently arose from missing outcome data and from selection of the reported result. Figure 2 presents a traffic-light visualization of risk of bias judgments across studies and outcomes; detailed RoB assessments for each outcome from every included study (result-level assessment) are provided in Table S4.

**Figure 3:**
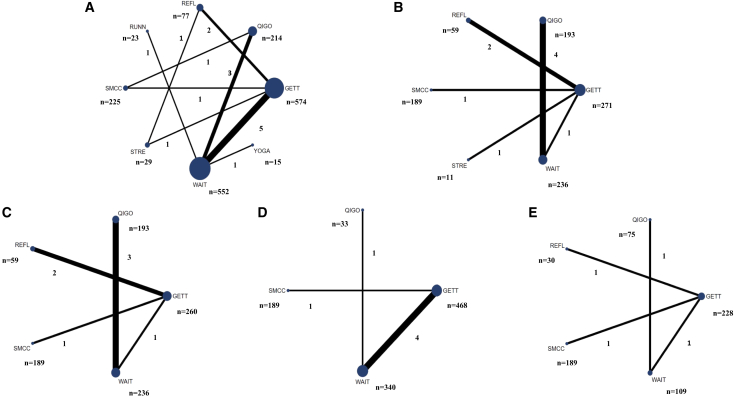
Network plots at the end of treatments Network plot of eligible direct comparisons for (A) fatigue, (B) depression, (C) anxiety, (D) physical function, and (E) sleep quality after treatments. Different instruments were used across trials, but outcome data were harmonized to the most widely applied scales: fatigue (CFQ, range 0–33, lower scores indicate less fatigue), depression (HADS-D, range 0–21, lower better), anxiety (HADS-A, range 0–21, lower better), physical function (SF-36 PF, range 0–100, higher better), and sleep quality (PSQI, range 0–21, lower better). Assessments were conducted around 12 weeks (IQR, 9–18 weeks). In the plots, the numbers and width of each line reflects the number of trials directly comparing two interventions, and the size of each node represents the number of randomized participants assigned to that intervention. REFL, relaxation/flexibility; QIGO, Qigong; GETT, graded excise therapy; WAIT, waiting list; STRE, strength/resistance training; SMCC, Specialist medical care; RUNN, running.

In the main text, we presented beneficial interventions for managing CFS, based on findings from the network meta-analysis, using the waitlist as the reference comparator for all subsequent estimates. The geometry of the treatment networks at the end of treatments is shown in Figure 3. Network density varied by outcome: fatigue had the most densely connected network, while physical function and sleep quality had sparser networks, with most evidence derived from a few direct comparisons. Across all outcomes, GET, Qigong, SMC, and waitlist were the most frequently studied interventions and served as central hubs, whereas interventions such as Yoga, relaxation/flexibility, running, and strength/resistance training were less frequently studied and typically linked through GET or waitlist. The uneven distribution of trials across comparisons indicates potential variation in the certainty of evidence between intervention pairs. Closed loops were observed only in the fatigue network, which enabled consistency checks between direct and indirect estimates. In contrast, the networks for depression, anxiety, sleep quality, and physical function were largely tree-shaped with very limited closed loops.

**Figure 4:**
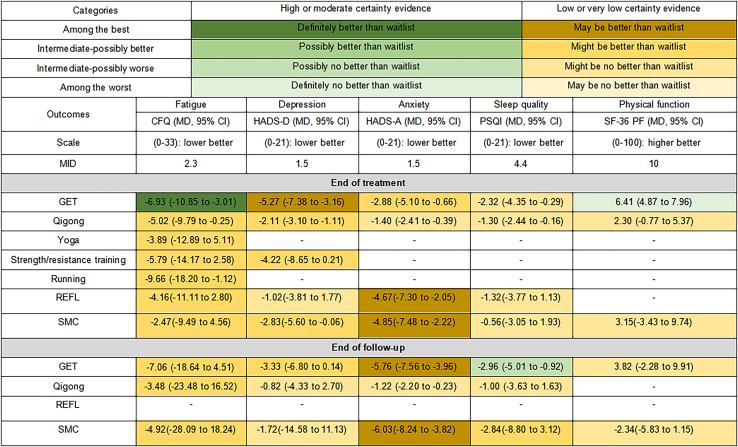
Summary of findings from the NMA compared with waitlist We classified the overall certainty of evidence into four categories: “very low,” “low,” “moderate,” or “high” using the Grading of Recommendations Assessment, Development, and Evaluation (GRADE) framework. We rated down for imprecision when the 95% CI crossed MID. We rated our certainty of the outcomes and classified interventions into the following four categories: 1) among the best: the intervention was superior to waitlist alone (the mean effect size exceeding the MID and the 95% CI not crossing the MID threshold); 2) intermediate-possibly better: the intervention was possibly superior to waitlist (the mean effect size exceeding the MID and the 95% CI crossing the threshold); 3) intermediate-possibly worse: the intervention was possibly not superior to waitlist (the mean effect size less than the MID and the 95% CI crossing the threshold); 4) among the worst: the intervention was not superior to waitlist (the mean effect size less than the MID and the 95% CI not crossing the threshold). The categories indicate whether the effect is clinically significant, while the certainty of evidence shows how reliable the effect is. CFQ, chalder fatigue scale questionnaire; HADS-D, hospital anxiety and depression scale-depression; HADS-A, hospital anxiety and depression scale-anxiety; SF-36 PF, short form 36-questionnaire-physical function; PSQI, sleep quality to the 21-point; MID, minimally important difference; MD, mean difference; CI, confidence interval.

Network plots for each outcome at the end of follow-up are shown in Figure S4. League tables for all comparisons at the end of treatments and follow-up are presented in Figure S1, respectively. Network forest plots are available in Figure S2, and heterogeneity estimates for all pairwise comparisons are reported in Figure S3. No evidence of publication bias was detected at the end of treatments or follow-up, as detailed in Table S5. The certainty of evidence for direct, indirect, and network estimates at both time points is provided in Table S6. In addition, Figure 4 shows the categorization of ET from among the best to among the worst when compared with waitlist for five outcomes, together with certainty of evidence and the magnitude of comparative effectiveness. Figure 4 also reports effectiveness both at the end of treatment and at follow-up.

### Meta-analysis of outcome indicators

#### Fatigue

Sixteen trials^16^^,^^18^^,^^26^^,^^30^^,^^32^^,^^33^^,^^34^^,^^36^^,^^37^^,^^38^^,^^39^^,^^42^^,^^46^^,^^48^ involving 1,867 participants evaluated 5 types of exercise interventions for fatigue at the end of treatments. Three trials used the chalder fatigue scale,^30^^,^^34^^,^^36^ while others were harmonized to this measure. All interventions were associated with reductions in fatigue. GET showed relatively superior short-term effects compared with waitlist (mean difference [MD]: −6.93, 95% confidence interval [CI]: −10.85 to −3.01, moderate certainty), with both the point estimate and CI exceeding the minimally important difference (MID). Qigong (MD: −5.02, 95% CI: −9.79 to −0.25, low certainty evidence), running (MD: −9.66, 95% CI: −18.20 to −1.12; very low certainty) and strength/resistance training (MD: −5.79, 95% CI: −14.17 to 2.58, very low certainty), and Yoga (MD: −3.89, 95% CI: −12.89 to 5.11, very low certainty) may be moderately effective, though their CIs overlapped the MID, indicating uncertainty in clinical relevance.

At the end of follow-up, five trials (*n* = 1,156)^30^^,^^31^^,^^39^^,^^41^^,^^43^ assessed 2 exercise interventions. GET (MD: −7.06, 95% CI: −18.64 to 4.51 low certainty) and Qigong (MD: −3.48, 95% CI: −23.48 to 16.52, very low certainty) may offer modest benefits, but wide CI and MID overlap limit confidence in sustained effectiveness.

#### Depression

Eight trials (*n* = 959)^29^^,^^30^^,^^32^^,^^33^^,^^36^^,^^39^^,^^43^^,^^48^ assessed depression at the end of treatments using the hospital anxiety and depression scale-depression (HADS-D) or converted equivalents. GET yielded the largest improvement (MD: −5.27, 95% CI: −7.38 to −3.16; low certainty). Qigong (MD: −2.11, 95% CI: −3.10 to −1.11) and strength/resistance training (MD: −4.22, 95% CI: −8.65 to 0.21), both with very low certainty evidence, showed potential but imprecise effects due to MID overlap.

At the end of follow-up, five trials (*n* = 957)^16^^,^^29^^,^^30^^,^^39^^,^^43^ using HADS-D indicated that GET (MD: −3.33, 95% CI: −6.80 to 0.14, low certainty) may confer moderate benefit, though CI again overlapped the MID.

#### Anxiety

Seven trials (*n* = 937)^29^^,^^30^^,^^32^^,^^36^^,^^39^^,^^43^^,^^48^ measured anxiety at the end of treatments using hospital anxiety and depression scale-anxiety (HADS-A). GET showed potential benefit (MD: −2.88, 95% CI: −5.10 to −0.66, low certainty), though the MID was not surpassed with confidence.

At the end of follow-up, four trials (*n* = 662)^29^^,^^30^^,^^39^^,^^43^ reported a more pronounced effect of GET (MD: −5.76, 95% CI: −7.56 to −3.96, low certainty), with both the point estimate and CI exceeding the MID, indicating a likely sustained and clinically meaningful reduction.

#### Physical function

At the end of treatments, six trials (*n* = 1,188)^16^^,^^18^^,^^34^^,^^37^^,^^39^^,^^43^ assessed physical function using the SF-36 PF or converted alternatives. Neither GET (MD: 6.41, 95% CI: 4.87 to 7.96, moderate certainty) nor Qigong (MD: 2.30, 95% CI: −0.77 to 5.37, low certainty) were arduous to produced clinically meaningful improvement, with estimates below and not overlapping the MID.

At the end of follow-up, five trials (*n* = 1,052)^31^^,^^35^^,^^39^^,^^41^^,^^43^ confirmed minimal benefit, with GET yielding an MD of 3.82 (95% CI: −2.28 to 9.91, low certainty), still below the MID threshold.

#### Sleep quality

Four trials (*n* = 631)^30^^,^^32^^,^^39^^,^^43^ evaluated sleep quality at treatment end using the PSQI or converted measures. GET (MD: −2.32, 95% CI: −4.35 to −0.29) and Qigong (MD: −1.30; 95% CI: −2.44 to −0.16), both low certainty, arduous to demonstrate clinically relevant effects, with point estimates and CI below the MID.

At the end of follow-up, four trials (*n* = 845)^16^^,^^30^^,^^39^^,^^43^ showed GET (MD: −2.96, 95% CI: −5.01 to −0.92, moderate certainty) and Qigong (MD: −1.00, 95% CI: −3.63 to 1.63, low certainty) may have a modest but likely non-beneficial effect.

#### Sensitivity and subgroup analyses

Of the predefined effect modifiers (age, sex, RoB, and diagnostic criteria), only diagnostic criteria could be analyzed due to data limitations. Subgroup analysis revealed no evidence of effect modification by diagnostic criteria, with low credibility due to being based on between-trial comparisons, involving a very small number of studies in the smallest subgroup, a high likelihood that the findings were due to chance, and the testing of multiple effect modifiers (four) without adequate consideration of multiplicity in the statistical analysis. Detailed results at the end of treatment and follow-up are presented in Tables S7 and S8. Sensitivity analyses using standardized mean differences (SMDs) without scale conversion (restricted maximum likelihood [REML] and DerSimonian-Laird [DL]) were directionally consistent with the harmonized MD primary analysis; for example, for fatigue at end of treatment, GET vs. waitlist yielded SMDs of −0.64 (95% CI −1.06 to −0.23; REML) and −0.64 (95% CI −1.03 to −0.25; DL), closely aligning with the primary MD estimate of −6.93 (−10.85 to −3.01) (Table S9). Sensitivity analyses confirmed the robustness of the primary findings, with consistent results across all approaches. Detailed sensitivity analysis results are provided in Table S9.

## Discussion

This systematic review and network meta-analysis synthesized evidence from 20 RCTs assessing ET for CFS. GET consistently demonstrated relatively superior short-term effects and clinically meaningful improvements in fatigue, depression, and anxiety. While other modalities, such as Qigong, Yoga, and strength/resistance training, showed some potential benefits, their effects were generally imprecise and did not exceed the MID thresholds with confidence. The effects of GET appeared partially sustained at the end of follow-up for fatigue and anxiety, whereas improvements in physical function and sleep quality remained below clinically meaningful thresholds, indicating limited long-term impact. These findings suggest the moderate short-term efficacy of GET, while highlighting considerable uncertainty regarding its long-term effectiveness and the clinical relevance of alternative interventions due to low-certainty evidence and overlapping confidence intervals.

Several methodological strengths enhance the credibility and clinical utility of our findings. First, we conducted a comprehensive network meta-analysis covering a broad spectrum of ET for CFS, incorporating both direct and indirect comparisons to improve study inclusion and analytical robustness. Second, outcome harmonization was achieved by rescaling data to common reference instruments, enabling cross-study comparability. Third, we used a minimally contextualized ranking approach based on MID thresholds and systematically assessed the certainty of evidence using the Grading of Recommendations Assessment, Development, and Evaluation (GRADE) framework. These strategies collectively enhance the transparency, interpretability, and applicability of our results in clinical decision-making.

Despite these strengths, the clinical use of GET remains highly debated. Although our analysis indicates significant benefits of GET both immediately post intervention (MD: −6.93) and at long-term follow-up (MD: −7.06), its broader endorsement is controversial. The UK National Institute for Health and Care Excellence 2021 guideline underscores that no curative treatment for CFS exists and instead promotes patient-led symptom management. It explicitly advises against recommending structured exercise programs with fixed incremental progression (e.g., GET), particularly those based on deconditioning or activity-avoidance models. This position has been challenged by Professor Flottorp, who criticizes the guideline for its lack of robust evidence and methodological shortcomings, including issues in diagnostic definitions, study selection, data analysis, and evidence grading.^49^ Meanwhile, the German Institute for Quality and Efficiency in Health Care supports the use of GET for patients with mild to severe CFS.^50^ A more fundamental source of controversy relates to safety concerns. Professor Vink, for instance, has highlighted critical flaws in the supporting evidence base,^51^ such as inadequately controlled study designs, reliance on subjective fatigue measures in unblinded trials, neglect of objective outcomes like the 6-min walk test, and insufficient reporting of adverse events, with some patients experiencing symptom exacerbation. These divergent views underscore the need for rigorous, transparent evaluations of GET that integrate both subjective and objective outcomes, systematically assess harms as well as benefits, and emphasize the importance of professional oversight by trained physiotherapists to ensure individualized and safe implementation. One possible explanation for the relatively superior short-term effects of GET is its structured and progressive nature, which may help reduce physical deconditioning and fear-avoidance behaviors, thereby improving patients’ confidence and adherence to activity.

Qigong, as another promising intervention, showed some therapeutic potential in our network analysis. Specifically, it was associated with modest improvements in fatigue (MD: −4.38) and depressive symptoms (MD: −2.11); however, neither effect reached the MID threshold, and the certainty of evidence was rated low. These limitations may stem from the small number of Qigong-related studies, sparse indirect evidence within the network structure, and a high risk of bias in key methodological domains. Nonetheless, the previous systematic review indicated that Chinese mind-body therapies, including Tai Chi and Qigong, may alleviate fatigue, anxiety, and depression, supported by moderate-certainty evidence.^22^ These findings suggest that, while Qigong holds potential, further high-quality trials are needed to substantiate its clinical value.

These findings have important implications for key stakeholders. For patients, they suggest that certain exercise-based interventions, particularly GET, may offer short-term symptom relief, though potential risks and uncertainties underscore the need for individualized and carefully monitored approaches. For healthcare providers, the results highlight the importance of shared decision-making, incorporating patient preferences, and tailoring therapy intensity to avoid overexertion. For guideline developers and policy-makers, our synthesis underscores the conflicting evidence base for GET and the limited certainty of alternative modalities, emphasizing the need for transparent, patient-centered trials to inform future recommendations. Although the point estimate for GET at long-term follow-up (MD: −7.06) was close to that observed immediately post treatment (MD: −6.93), the wide confidence interval (−18.64 to 4.51) indicates substantial imprecision and precludes firm conclusions regarding sustained benefit. This variability may reflect differences in adherence after the intervention period, with some participants continuing structured exercise and others showing poor compliance. However, as adherence and attrition were insufficiently reported, this interpretation remains speculative. These limitations highlight the need for future studies to systematically capture adherence and attrition data in order to clarify their influence on long-term outcomes.

Future clinical trials should aim to establish the absolute effects of promising modalities such as Qigong and Yoga by conducting high-quality RCTs with longer follow-up periods, prespecified timelines, and rigorous monitoring of adherence. Moreover, trials should stratify participants by disease severity (e.g., mild vs. moderate/severe CFS) to clarify whether specific subgroups may derive greater benefit or experience higher risk from interventions such as GET. To strengthen the evidence base and enhance applicability to clinical practice and guideline development, studies should also minimize missing data and ensure comprehensive reporting of all predefined outcomes. Such efforts will be critical to reduce uncertainty, improve comparability across studies, and ultimately inform evidence-based, patient-centered recommendations.

### Conclusion

In conclusion, we found that GET showed relatively superior short-term effect, with clinically meaningful improvements in fatigue, depression, and anxiety among patients with CFS. For fatigue at the end of treatment, the certainty of evidence was moderate, with partial maintenance of effects at follow-up supported by lower certainty. Other exercise modalities, including Qigong, Yoga, strength/resistance training, and running, showed possible benefits compared with waitlist controls, but the supporting evidence was of low/very low certainty. Overall, these findings suggest that exercise-based interventions may contribute to symptom management in CFS, although the current evidence base remains limited by methodological shortcomings and low certainty. Future research should prioritize well-designed head-to-head trials, incorporate both subjective and objective outcomes, and develop individualized, precision-based exercise prescriptions that can be feasibly translated into clinical practice.

### Limitations of the study

There were several limitations in our systematic review. First, we restricted inclusion to trials examining the isolated effects of exercise interventions and excluded gray literature, which may have introduced publication bias. However, no evidence of publication bias was detected at the end of treatments or follow-up, as assessed by funnel plots and Egger tests (Table S5). Second, our analyses primarily focused on the type of exercise and did not explore the influence of intervention setting, session duration, or training volume (a proxy for intensity) on outcomes, which may modulate the effects of exercise on fatigue and related symptoms. Third, although we discussed the aims and design of this study with members of the public and several authors have personal experience of living with CFS, patients or the public were not formally involved in the conduct, reporting, or dissemination of this review. Future research should prioritize elucidating dose-response relationships and refining precision-based exercise protocols tailored to individual patient profiles.

## Resource availability

### Lead contact

Further information and requests for resources should be directed to and will be fulfilled by the lead contact, Min Fang (fm-tn0510@shutcm.edu.cn).

### Materials availability

This study did not generate new unique reagents.

### Data and code availability

The data utilized in this meta-analysis originated from published studies; no new data or codes were employed. All data are described in the key resources table section. Any additional information required to reanalyze the data reported in this paper is available upon request from the lead contact.

## Acknowledgments

We would like to thank the Shanghai Municipal Central-Local Joint S&T Development Fund Program (YDZX20243100002004), the Shanghai Key Laboratory of Tuina Techniques on Musculoskeletal Disorders (24dz2260200), Three Year Action Plan for Shanghai to Further Accelerate the Inheritance, Innovation and Development of Traditional Chinese Medicine (ZY(2025–2027)-3-1-1), a special project of 10.13039/501100019978Shanghai Pudong new area health commission (PW2023E-01), and Shuguang Hospital Open Competition Program (SGYYJBGS-001).

## Author contributions

M.F. and L.K. designed the study; S.W. and J.R. performed the literature screening; Z.L. extracted the data; J.R. and S.Z. performed the risk of bias assessment; S.F. and J.R. analyzed or interpreted the data; S.Z. and Z.L. wrote and revised the paper; S.W. and J.R. carried out the final revision.

## Declaration of interests

The authors declare that there is no conflict of interest regarding the publication of this paper.

## STAR★Methods

### Key resources table

**Table:** 

REAGENT or RESOURCE	SOURCE	IDENTIFIER
**Deposited data**		

International prospective register of systematic reviews	PROSPERO	https://www.crd.york.ac.uk/PROSPERO/
Studies for meta-analysis	MEDLINE, Embase, Web of Science, PsycINFO, Cochrane Library, CNKI, VIP databases, SinoMed, and Wanfang	The studies included are referenced in Table 1.

**Software and algorithms**		

R Software 4.5.1	R Foundation for Statistical Computing	https://www.r-project.org
EndNote X9.1	Clarivate Analytics	https://endnote.com/
STATA SE15.0	StataCorp LLC	https://www.stata.com

### Experimental model and subject details

Our study does not use experimental models typical in the life sciences.

### Method details

#### Literature search

The search strategy was applied to nine databases (MEDLINE, Embase, Web of Science, PsycINFO, Cochrane Library, China National Knowledge Infrastructure, China Wanfang Data Information, Chinese biomedical literature service system, and Weipu Database for Chinese Technical Periodicals) from their inception until February 19, 2025, without restrictions on language or publication status. We screened additional eligible studies identified from the reference lists of related systematic reviews and guidelines. No restrictions (language or publication status) were applied. Search terms were developed using a combination of free-text keywords and MeSH terms. A sample search structure was: (“chronic fatigue syndrome” MeSH OR “myalgic encephalomyelitis” OR “CFS” OR “ME/CFS”) AND (“exercise therapy” MeSH OR “physical activity” OR “aerobic training” OR “resistance training” OR “strength training” OR “graded exercise therapy” OR “yoga” OR “tai chi” OR “qigong”). The full electronic search strategy for all databases was provided in Table S1. Specifically, two reviewers (SHZ and ZXL) independently performed the search and screening. Discrepancies were resolved through discussion, with arbitration by a third reviewer (JR) if necessary.

#### Eligibility criteria

We defined eligibility criteria using the PICO framework: 1) Population (P): We included randomized controlled trials (RCTs) enrolling participants diagnosed with CFS, based on recognized diagnostic criteria including CDC-1988, CDC-1994, the Canadian Consensus Criteria (CCC-2003 or CCC-2010R), and NICE-2007/2021; 2) Intervention (I): The intervention of interest was ET, conceptualized as a purposeful, structured, and repetitive form of physical activity aimed at improving or maintaining one or more components of physical health.^52^^,^^53^ Included modalities encompassed GET, Qigong, Yoga, strength/resistance training, and running; 3) Comparator (C): Acceptable control conditions included waitlist, specialist medical care, and relaxation/flexibility therapy; 4) Outcomes (O): The primary outcome was fatigue, assessed by validated scales (e.g., Chalder Fatigue Scale, CFQ). Secondary outcomes included depression (e.g., Hospital Anxiety and Depression Scale-Depression, HADS-D), anxiety (e.g., Hospital Anxiety and Depression Scale-Anxiety, HADS-A), sleep quality (e.g., Pittsburgh Sleep Quality Index, PSQI), physical function (e.g., Short Form 36-Questionnaire-Physical Function, SF-36 PF), and adverse events. Eligible studies were required to report outcomes assessed with validated instruments or provide detailed adverse event data. Trials lacking sufficient statistics to calculate effect sizes (mean and standard deviation, or convertible equivalents) for at least one prespecified outcome or adverse event were excluded from the quantitative synthesis. Two reviewers (SHZ and ZXL) independently screened studies according to these criteria using EndNote X9. Disagreements were resolved through discussion or, when necessary, consultation with a third reviewer (JR).

#### Data extraction

Two reviewers (SHZ and ZXL) independently screened full-text articles of all potentially eligible studies and assessed them for final inclusion. Reasons for exclusion were documented in Table S2. Discrepancies were resolved through team consensus. Data were extracted using a standardized data collection form that included study characteristics (first author, publication year, and country), participant characteristics (sample size, age, and genders), intervention protocols (exercise type, duration, and control types), and outcomes. All outcome data were collected as reported in the original publications. For trials with multiple related publications, we extracted data from the most comprehensive report and supplemented it with additional information from other related publications if they reported eligible outcomes not covered in the primary source. We ensured that the same outcome domain was not double-counted across publications. When necessary, we contacted the original study authors to clarify missing or unclear data. Any discrepancies in study selection or data extraction were discussed between the two reviewers, and unresolved disagreements were adjudicated by a third reviewer (JR).

#### Outcome measures

We adopted a minimally contextualized framework to interpret the results of the network meta-analysis by organizing interventions into four categories based on their relative effectiveness.^49^ Classification was based on whether the point estimate and its 95% CI exceeded the MID threshold. 1) among the best: the intervention was superior to waitlist alone (the mean effect size exceeding the MID and the 95% CI not crossing the MID threshold); 2) intermediate-possibly better: the intervention was possibly superior to waitlist (the mean effect size exceeding the MID and the 95% CI crossing the threshold); 3) intermediate-possibly worse: the intervention was possibly not superior to waitlist (the mean effect size less than the MID and the 95% CI crossing the threshold); 4) among the worst: the intervention was not superior to waitlist (the mean effect size less than the MID and the 95% CI not crossing the threshold). The categories indicate whether the effect is clinically significant, while the certainty of evidence shows how reliable the effect is.

#### Methodological quality assessment

Two reviewers (SHZ and ZXL) independently appraised study validity using the Cochrane Risk of Bias 2.0 (RoB 2) tool across five domains: bias arising from the randomization process^54^; bias due to deviations from intended interventions; bias due to missing outcome data; bias in measurement of the outcome; and bias in selection of the reported result. Each domain was rated as “high risk”, “low risk”, or “some concerns” according to the RoB 2.0 algorithm. Risk of bias assessments were summarized at the domain level for each outcome across studies, in accordance with the RoB 2 tool guidelines. Specifically, RoB was assessed only for outcomes that provided numerical data and were included in the quantitative synthesis. Outcomes without analyzable numerical results were not subjected to RoB assessment, as they did not contribute to the meta-analysis.^55^ Disagreements in risk of bias judgments were resolved through discussion or third-party adjudication (JR) when necessary.

#### Certainty of evidence

In addition, we assessed the overall confidence in the evidence using the Grading of Recommendations Assessment, Development, and Evaluation (GRADE) framework. The quality of direct comparisons was evaluated based on risk of bias, inconsistency, indirectness, and publication bias. We assessed the certainty of evidence for indirect estimates based on the lowest confidence rating among the contributing direct comparisons within the most influential first-order loop. Additional downgrading was applied when concerns regarding intransitivity were identified. For network estimates, the certainty of evidence was determined primarily by the dominant source, either direct or indirect, unless both contributed meaningfully and yielded consistent results, in which case the higher rating was retained. Further downgrading was considered in the presence of incoherence or imprecision. We evaluated imprecision in relation to established minimally important differences (MID) for each outcome. Specifically, we considered a 2.3-point reduction on the CFQ for fatigue, a 10-point improvement on the SF-36 PF for physical function, a 4.4-point change on the PSQI for sleep quality, and a 1.5-point difference on the HADS subscales for anxiety or depression.

#### Ethical considerations

No primary ethical review was required as analyses utilized deidentified, publicly available data from trials with original participant consent. Public stakeholders were not involved in study design or dissemination.

### Quantification and statistical analysis

To ensure comparability across studies using different instruments to assess similar outcomes (fatigue, sleep quality, anxiety, depression and physical function), we harmonized effect sizes by converting them to scores based on commonly used reference instruments for each outcome domain.^56^^,^^57^ The exact conversion formulas are provided in Table S10. This harmonization enabled the use of MID thresholds to judge clinical significance. As a robustness check, we also reanalyzed results using standardized mean differences (SMDs) without conversion, which yielded consistent findings (Table S9). Specifically, fatigue was standardized to the 33-point CFQ, sleep quality to the 21-point PSQI, physical function to the 100-point SF-36 PF, anxiety to the 21-point HADS subscale for anxiety (HADS-A), and depression to the 21-point HADS subscale for depression (HADS-D). We calculated the mean differences (MDs) with standard deviation (SD) for continuous outcomes, and results were analyzed separately for post-intervention and follow-up timepoints. If the means and SD were not directly reported, they were estimated from available data such as sample size, medians, interquartile range, or ranges, following established imputation methods.^58^^,^^59^^,^^60^^,^^61^ In studies that compared three or more interventions, if two arms belonged to the same intervention category as defined in this review, we combined these arms into a single group. The combined summary statistics (means, standard deviations, and sample sizes) were calculated using the formulae recommended in the Cochrane Handbook.^62^

For pairwise meta-analyses of direct comparisons, we employed DerSimonian-Laird random-effects models for meta-analysis of direct comparisons across all patient-important outcomes.^63^^,^^64^ Statistical heterogeneity was quantified using the *I*^2^ statistic, with interpretive thresholds as follows: 0%–40% was considered “might not be important”, 30%–60% as “moderate heterogeneity”, 50%–90% as “substantial heterogeneity”, and 75%–100% as “considerable heterogeneity”.^65^ To identify potential publication bias, we used Begger and Egger’s tests.^66^^,^^67^

The network meta-analysis was implemented using a frequentist approach based on a graph-theoretical approach via the netmeta package in R software. Treatment comparisons were illustrated through network diagrams created with the network plot command in Stata version 15.1 (StataCorp).^68^ We used the league tables and forest plots of the relative treatment effects to visualize comparisons of network estimations. To assess global inconsistency within the network, we employed Cochran’s Q test, while local inconsistency in each closed comparison loop was evaluated using the node-splitting method. To evaluate the transitivity assumption of indirect comparisons, we examined clinical and methodological similarities across study arms grouped by intervention type. Potential incoherence between direct and indirect estimates was further explored by comparing the overlap of point estimates and their corresponding 95% confidence intervals (CIs). We conducted predefined subgroup analyses to explore potential effect modifiers, including age (adults vs*.* children), sex (female vs*.* male), Rob (low vs*.* moderate/high) and diagnostic criteria (CDC vs*.* NICE vs*.* Oxford). We used ICEMAN tool to assess the credibility of subgroup effects.^69^ In addition, we also performed the sensitivity analyses to evaluate the robustness of the primary findings for the following variables: (1) separated the relaxation or flexibility group into relaxation or flexibility therapy, sham training; (2) excluded control conditions involving low-intensity active comparators with minimal or non-specific therapeutic effects-such as sham training, and relaxation or flexibility therapy; (3) excluded studies assessed as having high overall risk of bias; and (4) re-estimated effects without scale harmonization by computing SMDs and fitting random-effects network meta-analyses using restricted maximum likelihood (REML) and DerSimonian-Laird (DL) estimators, as a methodological sensitivity check against the harmonized MD primary analysis.

### Additional resources

The systematic review was reported following the Preferred Reporting Items for Systematic Review and Meta-analysis (PRISMA) 2020 statement and extension for network meta-analyses (PRISMA-NMA).^70^^,^^71^ The protocol was registered on PROSPERO (CRD420251029149; https://www.crd.york.ac.uk/PROSPERO/view/CRD420251029149).

## Supplemental information

Document S1. Figures S1–S4 and Tables S3–S5 and S7 and S10

Table S1. Search strategy in electronic databases Systematic literature review search terms and strategy, related to STAR Methods.

Table S2. Reasons for studies exclusion Studies excluded during full-text review with reasons for exclusion, related to STAR Methods.

Table S6. GRADE assessments GRADE assessments of the certainty of evidence for direct, indirect, and network estimates of short-term effects and follow-up outcomes, related to STAR Methods. a: Without considering the imprecision domain. b: Considering the logic in the network meta-analysis. RoB, risk of bias; GETT, graded exercise therapy; STRE, strength/resistance training; RUNN, running; QIGO, qigong; YOGA, yoga; SMCC, specialist medical care; REFL, relaxation/flexibility; WAIT, waitlist; NA, not applicable.

Table S8. ICEMAN Instrument for assessing the Credibility of Effect Modification Analyses (ICEMAN), related to STAR Methods. This table summarizes the credibility assessment of effect modification analyses in our study, evaluated using the ICEMAN tool. ICEMAN systematically rates potential effect modifiers across key methodological domains to determine overall credibility. Results support the robustness and interpretability of reported subgroup findings.

Table S9. Sensitivity analyses Sensitivity analyses of short-term effect and follow-up outcomes, related to STAR Methods. This table presents the results of sensitivity analyses conducted to evaluate the robustness of the short-term and follow-up effect estimates. The consistency of findings across different analytical assumptions confirms the reliability of the primary results.
