# Supplementary material for: Comparative efficacy of various exercise therapies for chronic fatigue syndrome: A systematic review and network meta-analysis
Source: iScience. 2025 Nov 21;28(12):114178. doi: 10.1016/j.isci.2025.114178 (PMC12744296; doi:10.1016/j.isci.2025.114178)
Supplement: Document S1. Figures S1–S4 and Tables S3–S5 and S7 and S10 [file mmc1.pdf]

**iScience, Volume *28***

## Supplemental information

# Comparative efficacy of various exercise therapies for chronic fatigue syndrome: A systematic review and network meta-analysis

**Zhongxin Liao, Suhong Zhao, Sitong Fang, Jun Ren, Shoujian Wang, Lingjun Kong, and Min Fang**

**Supplemental information titles and legends**

**Table S1. Search strategy in electronic databases.** Systematic literature review search terms and strategy, related to STAR Methods.

**Table S2. Reasons for studies exclusion.** Studies excluded during full-text review with reasons for exclusion, related to STAR Methods.

**Table S3. Description of intervention categories.**

**Table S4. Risk of bias assessments for included randomized controlled trials using the Cochrane RoB 2.0 tool, related to STAR Methods.** Cfs, chalder fatigue scale; CFQ, chalder fatigue questionnaire; HADS, hospital anxiety and depression scale; SF-36 PF, short form 36-questionnaire-physical function; FSS, fatigue severity scale; BAI, beck anxiety inventory; BDI, beck's depression index; JSS, jenkins sleep scale; MFI-20, the multidimensional fatigue inventory-20; NA, unreported.

**Figure S1. Network estimates of short-term effect and follow up, related to STAR Methods.** The figures show the relative effects of each intervention and waitlist (the column's treatment versus the row's treatment). The relative effects are measured as a mean difference for outcomes along with 95% CIs. The colour of each cell indicates the certainty of evidence according, the Grading of Recommendations Assessment, Development, and Evaluation (GRADE). Abbreviation: STRE = Strength/resistance training, RUNN = running, GET = Graded Exercise Therapy, QIGO = qigong, YOGA = yoga, SMC = specialist medical care, WAIT = waitlist, REFL = relaxation/flexibility, MD = Mean Differences , CI = Confidence Interval. A: Fatigue; B: Anxiety; C: Depression; D: Physical function; E: Sleep quality; F: Fatigue; G: Anxiety; H: Depression; I: Physical function; J: Sleep quality.

Certainty of evidence

High Moderate Low Very low

**Figure S2. Forest plot for the comparison of different exercise therapies interventions versus waitlist, related to STAR Methods.** Abbreviations: GETT = Graded Exercise Therapy, STRE = strength/resistance training, RUNN = running, QIGO = qigong, YOGA = yoga, SMCC = specialist medical care, REFL = relaxation/flexibility, WAIT = waitlist. A: Fatigue; B: Anxiety; C: Depression; D: Physical function; E: Sleep quality; F: Fatigue; G: Anxiety; H: Depression; I: Physical function; J: Sleep quality.

**Figure S3. Assessment of heterogeneity across meta-analyses, related to STAR Methods.** A: Fatigue; B: Anxiety; C: Depression; D: Physical function; E: Sleep quality; F: Fatigue; G: Anxiety; H: Depression; I: Physical function; J: Sleep quality.

**Figure S4. Network plots at end of follow-up, related to STAR Methods.** Abbreviations: RoB = Risk of Bias, GETT = Graded Exercise Therapy, STRE = strength/resistance training, RUNN = running, QIGO = qigong, YOGA = yoga, SMCC = specialist medical care, REFL = relaxation/flexibility, WAIT = waitlist. A: Fatigue; B: Anxiety; C: Depression; D: Physical function; E: Sleep quality.

**Table S5. Assessment of publication bias using Egger's test for short-term effects and follow-up outcomes, related to STAR Methods.** Abbreviations: GET = Graded exercise

therapy, QIGO = qigong, WAIT = waitlist.

**Table S6. GRADE assessments.** GRADE assessments of the certainty of evidence for direct, indirect, and network estimates of short-term effects and follow-up outcomes, related to STAR Methods. a: Without considering the imprecision domain. b: Considering the logic in the network meta-analysis. Abbreviations: RoB = Risk of Bias, GETT = Graded Exercise Therapy, STRE = strength/resistance training, RUNN = running, QIGO = qigong, YOGA = yoga, SMCC = specialist medical care, REFL = relaxation/flexibility, WAIT = waitlist, NA = Not Applicable.

**Table S7. Subgroup analyses by using different standardized diagnostic criteria, related to STAR Methods.** Abbreviations: GET = Graded exercise therapy, WAIT = waitlist, NA = Not Applicable. The global score of the Chalder Fatigue Scale, ranging from 0 to 33 with lower scores indicating better outcomes, was used to assess fatigue. A minimally important difference of 2.3 points was employed to evaluate improvements in fatigue. The subscale score of the Short Form 36-Questionnaire-Physical Function, ranging from 0 to 100 with higher scores indicating better outcomes, was used to assess physical function. A minimally important difference of 10 points was employed to evaluate improvements in physical function.

**Table S8. ICEMAN.** Instrument for assessing the Credibility of Effect Modification Analyses (ICEMAN), related to STAR Methods. This table summarizes the credibility assessment of effect modification analyses in our study, evaluated using the ICEMAN tool. ICEMAN systematically rates potential effect modifiers across key methodological domains to determine overall credibility. Results support the robustness and interpretability of reported subgroup findings.

**Table S9. Sensitivity analyses.** Sensitivity analyses of short-term effect and follow-up outcomes, related to STAR Methods. This table presents the results of sensitivity analyses conducted to evaluate the robustness of the short-term and follow-up effect estimates. The consistency of findings across different analytical assumptions confirms the reliability of the primary results.

**Table S10. Statistical conversion formulas for meta-analysis, related to STAR Methods.**

**Table S3. Description of intervention categories.**

| Intervention categories | Description |
|---|---|
| Graded exercise therapy (GET) | exercise in which the incremental increase in exercise was mutually set,including graded exercise (delivered by specialist therapists), and graded exercise self-help (self-help approaches based on GET principles). |
| Strength/resistance training | Exercise involving progressive overload of muscle groups through external resistance, including clinician-guided resistance training (structured programs delivered by physiotherapists or certified trainers with systematic load/volume progression), and self-managed resistance training (independently implemented regimens based on strength training principles, e.g., weight machines, free weights, resistance bands, or bodyweight exercises). The intensity, frequency, and resistance level are incrementally adjusted based on individual capacity and adaptation. |
| Running | A form of aerobic exercise involving sustained locomotion at varying speeds. |
| Qigong | A traditional Chinese mind-body practice combining slow movements, breath regulation, and focused intention to cultivate and balance Qi (vital energy), including teacher-guided qigong (structured routines led by certified masters or instructors, e.g., medical qigong, Ba Duan Jin [Eight Brocades], Wu Qin Xi [Five Animal Frolics], or Taoist/Buddhist lineage-based forms with precise alignment and energetic guidance), and self-practiced qigong (independently performed exercises using instructional videos, written manuals, or community-learned techniques, such as daily standing postures [Zhan Zhuang], meridian-tapping sequences, or simplified energy circulation practices). |
| Yoga | A holistic mind-body practice integrating physical postures, breath control, and meditation, including instructor-led yoga (structured sessions guided by certified teachers, e.g., Hatha, Vinyasa, Iyengar, or restorative yoga classes with tailored sequences and alignment cues), and self-guided yoga (independently practiced routines using digital platforms, books, or personal knowledge, such as home sessions, mindfulness flows, or targeted flexibility/mobility sequences). |

| | |
|---|---|
| Relaxation/flexibility | Relaxation:consists of techniques that aim to increase muscle relaxation (e.g.autogenic training, listening to a relaxation tape). Flexibility: includes stretches performed according to selected exercises given. |
| Specialist medical care | Includes disease explanation, general advice, self-help strategies, and symptomatic medication. |
| Waitlist | Keep their usual lifestyle and do not participate in any structured or proactive exercise programs during the study period. |

**Table S4. Risk of bias assessments for included randomized controlled trials using the Cochrane RoB 2.0 tool, related to STAR Methods.**

| Study ID | Outcome | Result | Randomization process | Deviations from intended interventions | Missing outcome data | Measurement of the outcome | Selection of the reported result | Overall Bias |
|---|---|---|---|---|---|---|---|---|
| Clark 2017 | Fatigue | Cfs | Low | Low | Low | Low | Low | Low |
| Clark 2017 | Physical function | SF-36 PF | Low | Low | Low | Low | Low | Low |
| Clark 2021 | Fatigue | Cfs | Low | Low | Low | Low | Low | Low |
| Clark 2021 | Physical function | SF-36 PF | Low | Low | Low | Low | Low | Low |
| Fulcher 1997 | Fatigue | Cfs | Low | Low | Low | Low | Some concerns | Some concerns |
| Ho 2012 | Fatigue | Cfs | Low | Low | High | Low | High | High |
| Ho 2012 | Physical function | SF-36 PF | Low | Low | High | Low | High | High |
| Jason 2007 | Anxiety | BAI | Some concerns | Low | Some concerns | Some concerns | Some concerns | Some concerns |
| Jason 2007 | Depression | BDI | Some concerns | Low | Some concerns | Some concerns | Some concerns | Some concerns |
| Moss-Morris 2005 | Fatigue | Cfs | Low | Low | Low | Low | Some concerns | Some concerns |
| Moss-Morris 2005 | Physical function | SF-36 PF | Low | Low | Low | Low | Some concerns | Some concerns |
| Oka 2014 | Fatigue | Cfs | Some concerns | Low | Low | Low | Some concerns | Some concerns |
| Powell 2001 | Fatigue | Cfs | Low | Low | Low | Low | Some concerns | Some concerns |
| Sutcliffe | Fatigue | FIS | Low | Low | Low | Low | Some | Some |

| | | | | | | | | |
|---|---|---|---|---|---|---|---|---|
| 2010 | | | | | | | concerns | concerns |
| Wallman 2004 | Fatigue | Cfs | Low | Low | Low | Low | Some concerns | Some concerns |
| Wallman 2004 | Anxiety | HADS | Low | Low | Low | Low | Some concerns | Some concerns |
| Wallman 2004 | Depression | HADS | Low | Low | Low | Low | Some concerns | Some concerns |
| Wearden 1998 | Fatigue | Cfs | Low | Low | Some concerns | Low | Some concerns | Some concerns |
| Wearden 2010 | Fatigue | Cfs | Some concerns | Low | Low | Low | Low | Some concerns |
| Wearden 2010 | Sleep | JSS | Some concerns | Some concerns | Low | Some concerns | Low | Some concerns |
| Wearden 2010 | Anxiety | HADS | Some concerns | Some concerns | Low | Some concerns | Low | Some concerns |
| Wearden 2010 | Depression | HADS | Some concerns | Some concerns | Low | Some concerns | Low | Some concerns |
| Wearden 2010 | Physical function | SF-36 PF | Some concerns | Some concerns | Low | Some concerns | Low | Some concerns |
| White 2011 | Fatigue | Cfs | Low | Low | Low | Low | Low | Low |
| White 2011 | Sleep | JSS | Low | Low | Low | Low | Low | Low |
| White 2011 | Anxiety | HADS | Low | Low | Low | Low | Low | Low |
| White 2011 | Depression | HADS | Low | Low | Low | Low | Low | Low |
| Zhao 2022 | Fatigue | Cfs | Some concerns | Low | Low | Low | Some concerns | Some concerns |
| Chen 2021 | Fatigue | MFI-20 | Some concerns | Low | Low | Low | Some concerns | Some concerns |
| Chan | Fatigue | Cfs | Some | Low | Low | Low | High | High |

| | | | | | | | | |
|---|---|---|---|---|---|---|---|---|
| 2013 | | | concerns | | | | | |
| Chan 2013 | Anxiety | HADS | Some concerns | Low | Low | Low | High | High |
| Chan 2013 | Depression | HADS | Some concerns | Low | Low | Low | High | High |
| Chan 2014 | Fatigue | Cfs | Some concerns | Low | Low | Low | High | High |
| Chan 2017 | Anxiety | HADS | Some concerns | High | Low | Low | High | High |
| Gordon 2010 | Fatigue | CFQ | Low | Some concerns | Low | Low | Low | Some concerns |
| Sharpe 2015 | Fatigue | CFQ | Low | Low | Low | Low | Low | Low |

**Figure S1. Network estimates of short-term effect and follow up , related to STAR Methods.**

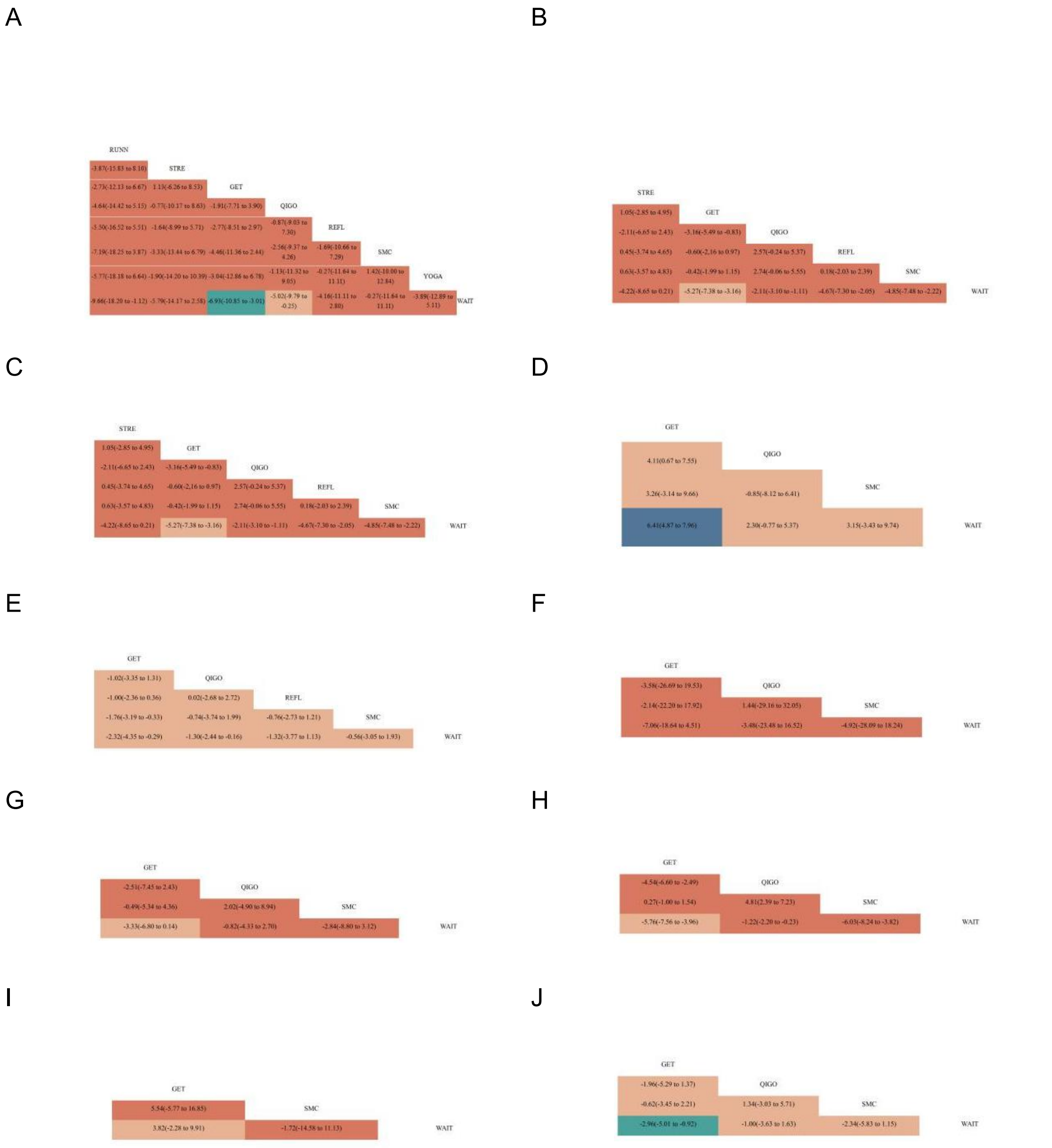

**Figure S2. Forest plot for the comparison of different exercise therapies interventions versus waitlist, related to STAR Methods.**

A

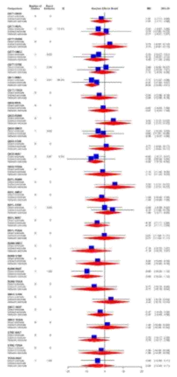

B

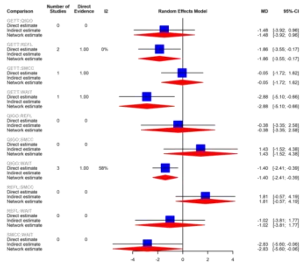

C

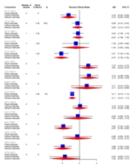

D

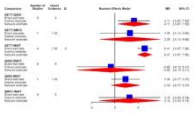

E

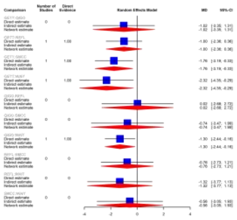

F

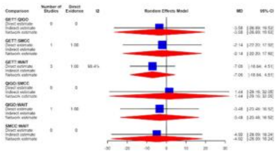

G

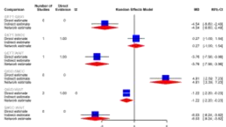

H

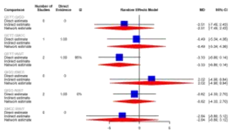

I

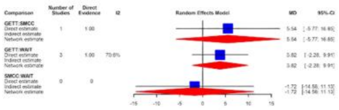

J

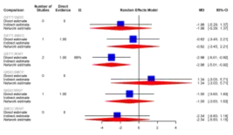

**Figure S3. Assessment of heterogeneity across meta-analyses, related to STAR Methods.**

A

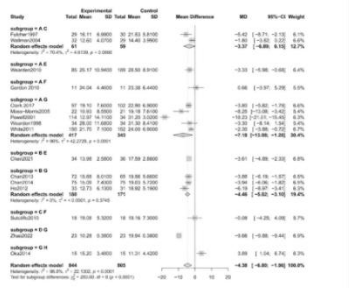

B

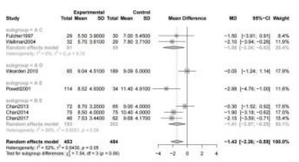

C

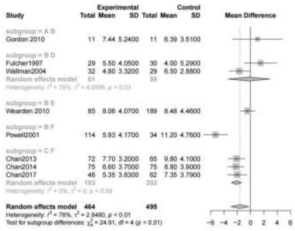


D

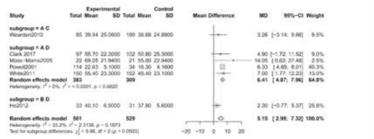

E

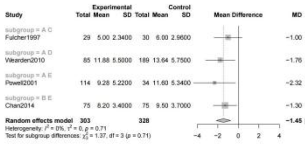


F

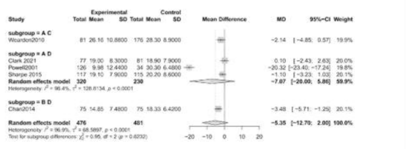

G

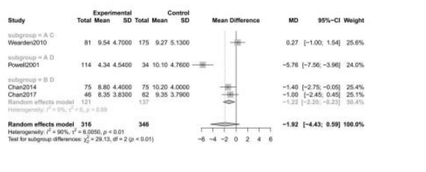


H

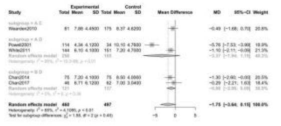

I

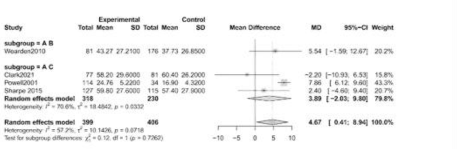


J

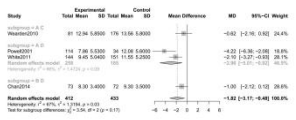

**Table S5. Assessment of publication bias using Egger's test for short-term effects and follow-up outcomes, related to STAR Methods.**

| | Comparison | Begg's Test (z) | Begg's Test (p) | Egger's test (z) | Egger's test (p) |
|---|---|---|---|---|---|
| **at short term** | | | | | |
| **Fatigue** | GET *v* WAIT | 0.73 | 0.462 | -0.03 | 0.975 |
| | QIGO *v* WAIT | 1.02 | 0.308 | -1.73 | 0.225 |
| **Anxiety** | QIGO *v* WAIT | 1.04 | 0.296 | 1.10 | 0.468 |
| **Depression** | QIGO *v* WAIT | 1.04 | 0.296 | -6.53 | 0.097 |
| **Physical function** | GET *v* WAIT | 0.34 | 0.734 | 6.17 | 0.025 |
| **Sleep quality** | GET *v* WAIT | -0.24 | 1.000 | 5.42 | 0.012 |
| **at the end of follow-up** | | | | | |
| **Fatigue** | GET *v* WAIT | 1.41 | 0.393 | 1.04 | 0.296 |
| **Physical function** | GET *v* WAIT | 16.71 | 0.038 | 1.04 | 0.296 |

**Table S7. Subgroup analyses by using different standardized diagnostic criteria, related to STAR Methods.**

| | Interventions | Main | CDC | NICE | Oxford |
|---|---|---|---|---|---|
| **at short-term** | | | | | |
| **Fatigue** | GET *v* WAIT | -6.93(-10.85 to -3.01) | -8.25(-13.08 to -3.42) | -3.80(-5.82 to -1.78) | -7.99(-18.16 to 2.19) |
| **Physical function** | GET *v* WAIT | 6.41(4.87 to 7.96) | 14.05(0.62 to 27.48) | 4.90(-1.72 to 11.52) | 6.39(4.79 to 7.99) |
| **at the end of follow-up** | | | | | |
| **Fatigue** | GET *v* WAIT | -7.07(-20.00 to 5.86) | NA | -0.10(-2.43 to 2.63) | -10.68(-29.51 to 8.16) |
| **Physical function** | GET *v* WAIT | 3.89(-2.03 to 9.80) | NA | -2.20(-10.93 to 8.78) | 6.23(1.33 to 11.13) |

**Figure S4. Network plots at end of follow-up, related to STAR Methods.**

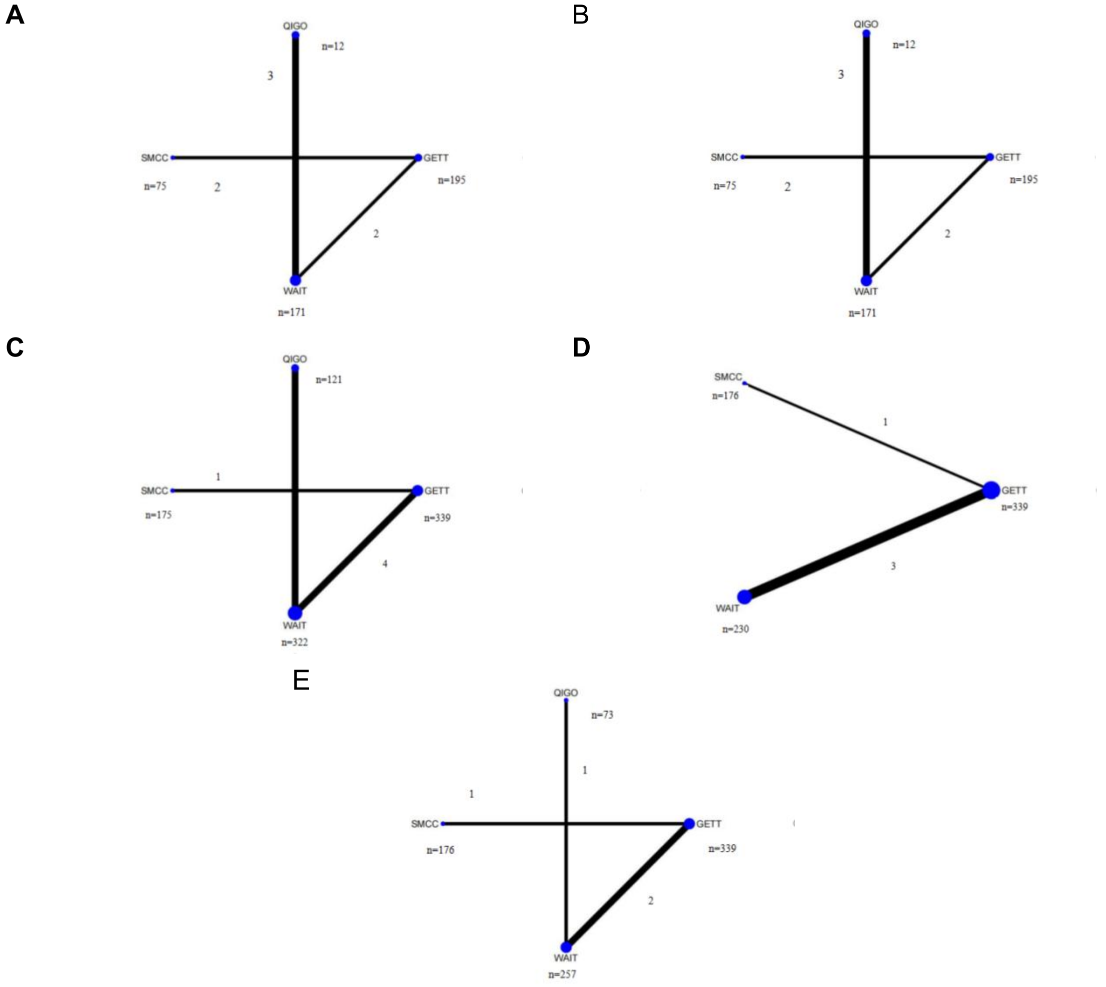

**Table S10. Statistical conversion formulas for meta-analysis, related to STAR Methods.**

A represents Beck Depression Inventory II (BDI-II) and B represents Hamilton Depression Rating Scale (HDRS). The BDI ranges from 0 to 63 and the HDRS from 0 to 54, where higher scores show the worse possible outcomes in the instruments. Then we use hypothetical values for one trial that report scores in HDRS units: MT = 4.50, SDT = 6.00, MC = 8.70, SDC = 7.40, where MT and MC represent the treatment and control means and SDT and SDC represent the treatment and control SDs. Thus, LA and LB (the lower limits of each instrument) are 63 and 54, respectively, and RA and RB (the range of each instrument) are -63 and -54, respectively.

The following formula was used to calculate the mean scores in BDI-II units:

*MAi = (MBi – LBi) * (RAi / RBi)+ LAi*

*MAT* = (4.50-54) * (-63/-54)+63=5.25.

*MAC* = (8.70-54) * (-63/-54)+63=10.15.

The following formula was to calculate the SDs in BDI-II units:

*SDAi = SDBi * (Rai / RBi)*

*SDAT* = 6*(-63/-54)=7

*SDAC* = 7.40*(-63/-54)=8.63.

Reference: Ebrahim S, Johnston BC, Akl EA, et al. Addressing continuous data measured with different instruments for participants excluded from trial analysis: a guide for systematic reviewers. J Clin Epidemiol. 2014;67(5):560-570. doi:10.1016/j.jclinepi.2013.11.014
